# Giant Intracranial Solitary Plasmacytoma

**DOI:** 10.4274/tjh.2016.0199

**Published:** 2017-12-01

**Authors:** Osman Kara, Tayfur Toptaş, Işık Atagündüz, Süheyla Bozkurt, Önder Şirikçi, Tülin Fıratlı Tuğlular

**Affiliations:** 1 Marmara University Faculty of Medicine, Department of Hematology, İstanbul, Turkey; 2 Marmara University Hospital Faculty of Medicine, Department of Pathology, İstanbul, Turkey; 3 Marmara University Hospital Faculty of Medicine, Department of Biochemistry, İstanbul, Turkey

**Keywords:** Plasmacytoma, Myeloma, Intracranial

A 41-year-old man presented with complaints of severe headache and vomiting during the last 5 days. Neurological and systemic examination revealed no abnormality. A mass of 112x49 mm, which occupied the left frontoparietal parenchymal region, was evident on the T_1_ sequence of magnetic resonance imaging (Figure 1). This homogeneous contrasted mass displaced the left lateral ventricle and caused a shift of the mid-verge of the brain. The mass was totally removed (Figure 2). Pathological examination revealed a plasma cell dyscrasia with lambda monoclonality.

Bone marrow biopsy was consistent with a clonal plasma cell accumulation of 5%. Two tiny M-protein peaks were detected on serum protein electrophoresis, which was compatible with immunoglobulin (Ig) G-and IgA-lambda monoclonal bands on serum immunofixation electrophoresis (Figure 3). There were no other plasmacytomas or lytic lesions detected with positron emission tomography-computed tomography imaging. The patient was diagnosed with a solitary plasmacytoma and treated with radiotherapy only. He had no complaints at the sixth month after diagnosis.

## Figures and Tables

**Figure 1:**
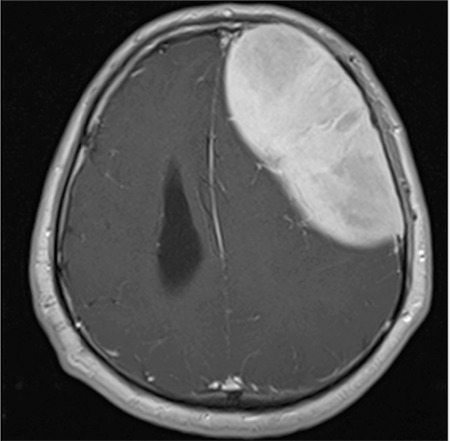
The mass occupied the left frontoparietal region, displacing the left lateral ventricle and causing a shift of the mid-verge of the brain.

**Figure 2:**
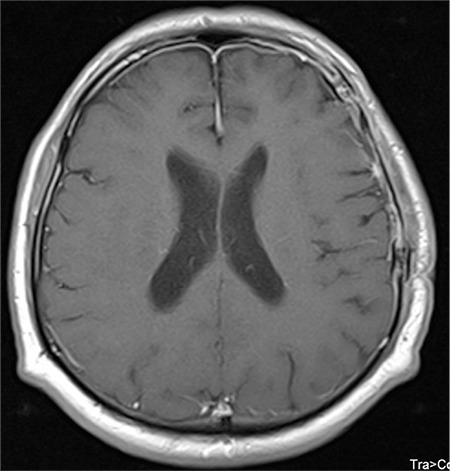
Postoperative cranial magnetic resonance imaging.

**Figure 3:**
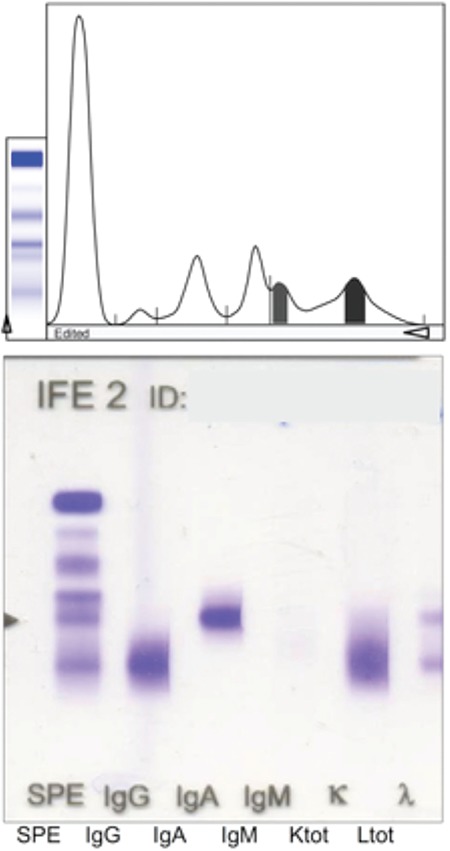
M-protein peaks on serum protein electrophoresis and monoclonal bands on immunofixation electrophoresis are depicted. Ig: Immunoglobulin.

